# How Carbon Source and Degree of Oligosaccharide Polymerization Affect Production of Cellulase-Degrading Enzymes by *Fusarium oxysporum* f. sp. *lycopersici*

**DOI:** 10.3389/fmicb.2021.652655

**Published:** 2021-03-26

**Authors:** Nasim Najjarzadeh, Leonidas Matsakas, Ulrika Rova, Paul Christakopoulos

**Affiliations:** Biochemical Process Engineering, Division of Chemical Engineering, Department of Civil, Environmental and Natural Resources Engineering, Luleå University of Technology, Luleå, Sweden

**Keywords:** *Fusarium*, induction, cellulase, cellooligosaccharides, xylooligosacharides, carbon source, degree of polymerization

## Abstract

Cellulases are a group of enzymes responsible for the degradation of cellulose, which is one of the most abundant polymers on Earth. The three main groups of cellulases are endoglucosidases, exoglucosidases, and β-glucosidases; however, the mechanism of induction of these enzymes remains poorly characterized. Cellooligosaccharides are among the main inducers of these enzymes in filamentous fungi, yet it is not clear how their degree of polymerization may affect the strength of induction. In the present study, we investigated the effect of different carbohydrate-based inducers, such as lactose, sophorose, cellooligosaccharides, and xylooligosacharides, characterized by different concentrations and degree of polymerization, on cellulases production by the fungus *Fusarium oxysporum* f. sp. *lycopersici*, which is one of the most studied lignocellulose degrading fungi with the ability to consume both cellulose and hemicellulose. Moreover, the effect of carbon source on cellulase induction was assessed by growing the biomass on sucrose or glycerol. Results showed a correlation between induction efficiency and the cellooligosaccharides’ concentration and size, as well as the carbon source available. Specifically, cellotetraose was a better inducer when sucrose was the carbon source, while cellobiose yielded a better result on glycerol. These findings can help optimize industrial cellulase production.

## Introduction

An increasing human population and limited natural resources have forced a search for new solutions. The strong dependency of industrial and technological advances on energy has led to an increase in fossil fuel consumption. Data show that crude oil is the most consumed fuel (39%), followed by coal (33%) and natural gas (33%) (Ritchie and Roser, 2020). As this trend leads to the accumulation of hazardous pollutants and emission of greenhouse gases, which result in global warming, alternative resources should be investigated. Studies show bio-based energy and products can decrease net CO_2_ emission to zero (Moshkin et al., 2005; Zhang et al., 2006).

Agriculture and wood industry residues are an abundant and cheap substrate for the production of chemicals and biofuel. Cellulose, which is a homopolymer composed of repeating units of anhydrocellobiose (Zhang et al., 2006), is the main component of plant biomass (Park et al., 2011; Obeng et al., 2017). It is produced by photosynthesis either by higher plants and algae or by non-photosynthetic organisms, including some bacteria and fungi, marine invertebrates, slime molds, and amoebae (Zhang and Lynd, 2004). Long chains and sheets of cellulose are linked by hydrogen bonds and van der Waal’s forces (Zhang et al., 2006). Cellulose has a recalcitrant structure and its β-glucosidic bonds have a half-life of 5–8 million years at 25°C (Wolfenden and Snider, 2001; Zhang et al., 2006). This rigidity is an obstacle for efficient industrial use of cellulose.

Cellulose can be hydrolyzed either chemically or enzymatically. Whereas chemical hydrolysis requires harsh conditions and dilute mineral acids (Panagiotou et al., 2005), enzymatic hydrolysis is a milder process with higher glucose yield (Mielenz, 2001). Enzymatic conversion of cellulose to fermentable sugars is the most complicated part of the process (Donohoe and Resch, 2015). Cellulases, which are responsible for cellulose degradation, can be either cell-bound or free enzymes (Zhang and Zhang, 2013). They are classified in 13 families of glycoside hydrolases (1, 3, 5, 6, 7, 8, 9, 12, 26, 44, 45, 51, and 48) based on similarities between amino acid sequences and crystal structures (Schu, 2000; Zhang and Zhang, 2013). Exoglucanase (EC 3.2.1.91), endoglucanase (EC 3.2.1.4), and β-glucosidase (EC 3.2.1.21) are the three main groups of hydrolytic enzymes that collaborate with auxiliary enzymes, such as lytic polysaccharide mono-oxygenases and phosphorolytic enzymes (in some bacteria), to disintegrate cellulose (Eriksson et al., 1990; Zhang et al., 2006). Endoglucanases randomly hydrolyze β-1,4-glucosidic bonds of cellulose chains and produce chain ends, where exoglucanase can act to release glucose or cellobiose. This rate-limiting step results in the release of variously polymerized soluble sugars from the surface of solid substrate into the liquid phase. Finally, β-glucosidases hydrolyze the released cellobiose and prevent end-product inhibition (Zhang et al., 2006).

Cellulases account for around 20% of the enzyme market (Bhat, 2000; Singhania et al., 2010). They are the third most consumed enzymes in industry, and the growing demand for bioenergy and bio-based materials promises a bright future for cellulase production (Rani et al., 2010; Chandel et al., 2012; Yoon et al., 2014). One of the main hindrances to the use of cellulose in biorefinery is the costly large-scale production of cellulases (Zhang et al., 2006). Specifically, it is difficult to increase the volume of produced enzyme using cheap substrates or generate enzymes with higher stability and/or specific activity on solid substrates (Zhang et al., 2006). Based on estimates, nearly 25 cents of every dollar used to produce bioethanol are spent on enzyme production (Humbird et al., 2011; Huberman et al., 2016), with around 100 g of cellulase being consumed per gallon of ethanol (Zhang et al., 2006; Zhu and Zhang, 2009).

Cellulases exist in a variety of microorganisms, from yeast, bacteria, and fungi to protozoa, plants, and animals (Zhang and Zhang, 2013); however, their industrial production relies on bacterial or fungal fermentation (Yoon et al., 2014). Compared to bacteria, fungi possess more advanced cellulase systems (Cen and Xia, 1999). Moreover, owing to their exceptional ecological, biological, and morphological plasticity, they can adapt to different environments and resources (Selbmann et al., 2013). As the complete saccharification of lignocellulosic biomass is time-consuming and needs a high load of degrading enzymes (around 30–50 mg per gram of crystalline cellulose), only fungi can meet such requirements (Ali et al., 2016).

*Fusarium oxysporum* is a soil-born plant pathogen responsible for vascular wilt disease in various crops, as well as mycotoxin contamination of human and animal food (Pathology, 2003). It is capable of producing several cell-wall degrading enzymes and is the most studied species in the *Fusarium* genus regarding lignocellulose degradation (Agrios, 2005). Its importance derives from its applicability in consolidated bioprocessing, whereby enzyme production, biomass hydrolysis, and fermentation of hexoses and pentoses to ethanol can be achieved in a single step (Panagiotou et al., 2003; Xu et al., 2009; Ali et al., 2016). Several endoglucanase genes (located on chromosomes 3, 12, 11, 4, 8, 7), β-glucosidase genes (located on chromosomes 5, 11, 1, 8, 12, 9, 7, 13), and one exoglucanase gene (located on chromosome 1) are deposited with NCBI for *Fusarium oxysporum* f. sp. *lycopersici*. Three endoglucanase enzymes from *F. oxysporum* have been characterized; one of them has a molecular weight of only 23.2 kDa, which facilitates penetration through cellulosic fibers (Kekos et al., 1995). Characterization of a β-glucosidase (Christakopoulos et al., 1991, 1995a), has revealed high β-glucosidase activity in this species that prevents end-product inhibition by cellobiose (Panagiotou et al., 2005). Studies on *F. oxysporum* strain 11C have indicated a highly efficient carbohydrate metabolism and ethanol production that depends on energy, protein, and sugar transport (Ali et al., 2014).

Despite numerous studies on the mechanism of cellulose degradation in this fungus, the machinery of cellulose sensing and enzyme induction/inhibition remains vague. This knowledge would allow researchers to modify and optimize biomass degradation by fungi, producing the desired product at high yields and with low byproducts. Most of the earlier investigations were based on the application of complex plant material, such as wheat straw and wheat bran, as carbon source with only few studies to use pure inducers. *Trichoderma reesei*, which is the most well known industrial cellulase producer, is influenced by substrate type (Els and Reese, 1956; Mandels and Reese, 1959; Mandels et al., 1961; Castro et al., 2014). Insoluble complex substrates, including plant biomass or crystalline cellulose, are good inducers of CAZymes (Alazi and Ram, 2018); however, they cannot be absorbed by mycelia and, therefore, they first need to be partially degraded to di- or monosaccharides such as cellooligosaccharides or their positional isomers such as sophorose (Alazi and Ram, 2018) prior to inducing cellulase genes. Sophorose, produced via transglycosylation during cellulose hydrolysis by an extracellular β-glucosidase, is the strongest cellulase inducer of *T. reesei* (Leite and Eveleight, 1989), but has no effect on *Aspergillus niger* (Gielkens et al., 1999) or *Phanerochaete chrysosporium* (Ulmer et al., 1984). Cellobiose induces cellulolytic enzymes in *Neurospora crassa*, *T. reesei*, and *Aspergillus* species but not in *P. chrysosporium* (Mandels and Reese, 1959; Vaheri et al., 1979; Chikamatsu et al., 1999; Znameroski et al., 2012), in which, instead, cellotriose and cellotetraose are excellent stimulants (Suzuki et al., 2010). In *Polyporus arcularius*, glucose, cellobiose, cellotriose, and cellotetraose repress enzyme production, while cellopentaose has the opposite effect (Ohnishi and Nagase, 2007). This variability shows the absence of a general rule and each species relies on specific inducers. Lactose, a cheap byproduct of some industries, has been used for cellulase production by *T. reesei* (Morikawa et al., 1995) and *Acremonium cellulolyticus* (Fang et al., 2008). Xylose can induce some cellulase genes in *A. niger* (Gielkens et al., 1999). In contrast, not many inducers have been documented in *F. oxysporum* and the mechanism of induction remains vague.

In this study, we aimed to identify effective inducers in *F. oxysporum* by examining some well-known inducers from other species, as well as cellooligosaccharides and xylooligosacharides with different degrees of polymerization and concentration. The obtained result facilitate toward better understanding of the mechanisms of induction mechanisms and the effect of the type of lignocellulosic biomass compounds on the induction of cellulolytic enzymes.

## Materials and Methods

### Microorganism and Preculture

All chemicals used in the current work were purchased from Sigma-Aldrich (St. Louis, MO, United States) unless otherwise stated. Sophorose, cellooligosaccharides, and xylooligosacharides were purchased from Megazyme, Bray, Ireland. *Fusarium oxysporum* f. sp. *lycopersici* (CBS 123668) was obtained from Centraalbureau voor Schimmelcultures (CBS; Utrecht, The Netherlands) and maintained on potato dextrose agar plates (39 g/L). The inoculum was prepared by cultivating the fungus for 2 days at 29°C and 190 rpm in 500 mL flasks containing 100 mL medium, which consisted of 20 g/L malt extract (MERCK; Kenilworth, NJ, United States) and 5 g/L yeast extract (MERCK).

### Enzyme Induction Experiment

For the induction experiment, the fungus was first grown on medium containing 0.2 M sodium phosphate buffer, 1 g/L KH_2_PO_4_, 0.3 g/L MgSO_4_⋅7H_2_O, 10 g/L (NH_4_)2HPO_4_, and 1% w/v sucrose as carbon source, with pH adjusted to 6.3. This medium was inoculated with 6% v/v of the preculture and then incubated at 29°C and 190 rpm for 2 days. Biomass was harvested through a sterile vacuum filter (0.45 μm; Sarstedt, Nümbrecht, Germany), aseptically washed twice with 0.9% saline solution, and inoculated in the same medium without the carbon source. The fungus was incubated for 1 day to deplete any residual sugar from the mycelium. Then, the inducers were added to the medium: 0.2 and 0.3% w/v lactose; 0.1, 0.2, and 0.3% w/v cellobiose; 0.1 and 0.2% cellotetraose; 0.1 and 0.2% cellohexaose; 0.1% w/v xylobiose; 0.1% w/v xylotetraose; 0.1% w/v xylohexaose; and 0,1% w/v sophorose. Induction was followed over 3 days, in which each sampling point was a separate flask and the whole culture was harvested to minimize sampling errors. The samples were filtered through glass-microfiber discs (grade MGA; Sartorius, Gottingen, Germany) and both the filtrate and the biomass were collected to determine secreted and cell-bound enzyme activity, respectively. To determine the influence of carbon source on enzyme induction, the same experiment was repeated using glycerol as carbon source and cellooligosaccharides as inducers.

### Enzymatic Activity Measurements

The activities of excreted enzymes including endoglucanase, exoglucanase, and extracellular β-glucosidase were measured using cell filtrates as a source of enzyme. For the endoglucanase assay, 200 μL of 1% w/v CMC solution and 50 μL of supernatant were incubated at 40°C for 15 min. The released reducing sugars were measured by the dinitrosalicylic acid method and compared to a glucose standard curve. The exoglucanase assay measured both the amount of released reducing sugars using the dinitrosalicylic method and 1% w/v microcrystalline cellulose solution (Avicel) as substrate, as well as the amount of p-nitrophenyl (pNP) released from 1 mM pNPC. Finally, β-glucosidase activity was measured using 1 mM solutions of p-nitrophenyl β-D-glucopyranoside (pNPG). When assaying exoglucanase and β-glucosidase activities with the pNP method, 100 μL of the enzyme solution was incubated with 900 μL of substrate at 40°C for 30 min. The reaction was stopped by the addition of 200 mL of 30% Na_2_CO_3_. All substrates were dissolved in phosphate-citrate buffer (pH 5). Cell-bound β-glucosidase activity was determined using 1.5 mg of air-dried intact cells as substrate and the pNPG assay. The activities are expressed as U/mL and U/mg for extracellular and cell-bound enzymes, respectively. All experiments were performed at least in three replicates, the results are presented as average and standard deviation.

### Cellooligosaccharides Uptake Experiment

Cellooligosaccharides separation and detection in the medium and intracellular compartment was done by high-performance anion-exchange chromatography. The induction experiment was repeated using cellooligosaccharides as inducers and sampling was carried out after 1, 3, 5, and 7 h. For intracellular detection, the biomass was collected after 1 h, dried in a towel, grounded by liquid nitrogen, and washed with 5 mL water. Then, it was boiled for 10 min, followed by centrifugation (10,000 rpm, 10 min) and the supernatant was labeled as fraction A. The pellet was resuspended in 3 mL water, boiled for 4 h, and centrifuged. The resulting supernatant was labeled as fraction B. All samples were analyzed by Dionex ICS-5000 (Thermo Fisher Scientific, Waltham, MA, United States) with a pulsed amperometric detector equipped with a disposable electrochemical gold electrode, using a CarboPac PA1 4 × 250 mm analytical column and a CarboPac PA1 4 × 50 mm guard column, at 30°C.

## Results

### Sucrose as Carbon Source

Our preliminary results using sucrose as carbon source during the production of fungal biomass show that xylooligosacharides and lactose have either no influence or cause a negligible increase in enzymatic activity compared to the control. In addition, sophorose, which is known as a good inducer for some fungi, was ineffective for the induction of most enzymes, except exoglucanase. Based on these findings, inefficient inducers were excluded from the rest of the study, which instead focused on cellooligosaccharides. Moreover, because no exoglucanase activity was detected using Avicel as a substrate, p-nitrophenyl-β-D-cellobioside (pNPC) was employed in its place.

**FIGURE 1:**
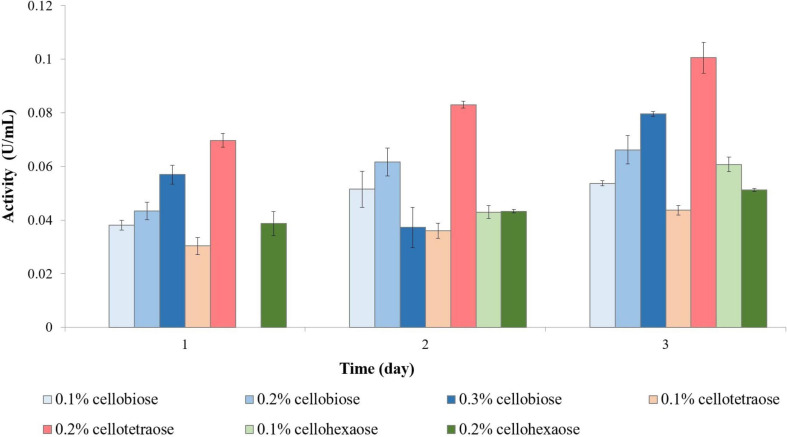
Endoglucanase activity of sucrose-grown biomass in the presence of different inducers and using CMC as substrate. No activity was detected in the presence of other inducers or the control sample.

The highest endoglucanase activity was induced by 0.2% cellotetraose (Figure 1). The concentration of inducer appeared to be an important factor, because endoglucanase inductionwas nearly double at 0.2% compared to 0.1% cellotetraose. The same result was observed with cellobiose, whereby induction was highest at 0.3%, followed by 0.2 and 0.1%. Similarly, doubling the cellohexaose concentration from 0.1 to 0.2% induced activity as early as on day 1. A comparison of the results shows that, besides concentration, the degree of polymerization of the inducer was essential for the induction process. The lower activity achieved by adding cellohexaose compared to cellotetraose and cellobiose indicates that the optimum degree of polymerization for an inducer was four.

**FIGURE 2:**
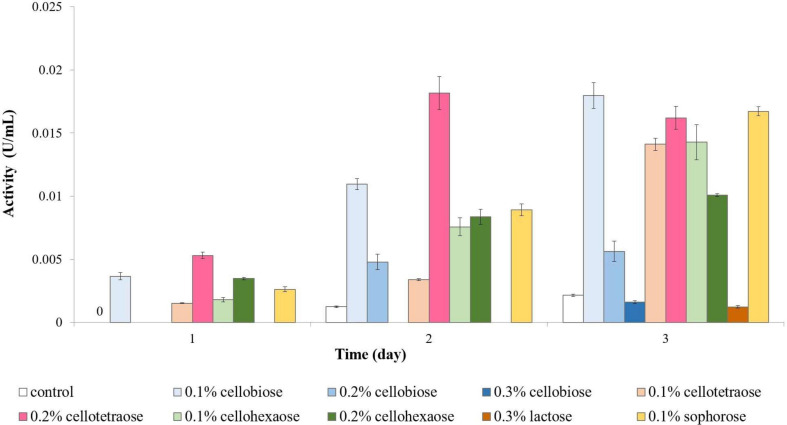
Exoglucanase activity of sucrose-grown biomass in the presence of different inducers and using pNPC as substrate. The sample containing 0.2% lactose failed to induce any activity.

Exoglucanase activity was highest with 0.2% cellotetraose, followed by 0.1% cellobiose and 0.1% sophorose. Inducer concentration was important for exoglucanase activity, albeit the effect differed between candidates. On the one hand, 0.2% was better than 0.1% cellotetraose; on the other hand, cellobiose and cellohexaose led to better outcomes at lower concentrations (Figure 2). For example, 0.1% cellobiose was effective already on day 1 and continued to be stronger than 0.2 or 0.3% cellobiose even in the following days, with 0.3% showing some induction only on day 3. Unlike endoglucanase, 0.1% sophorose caused a considerable increase in exoglucanase activity; whereas lactose had a negligible effect.

**FIGURE 3:**
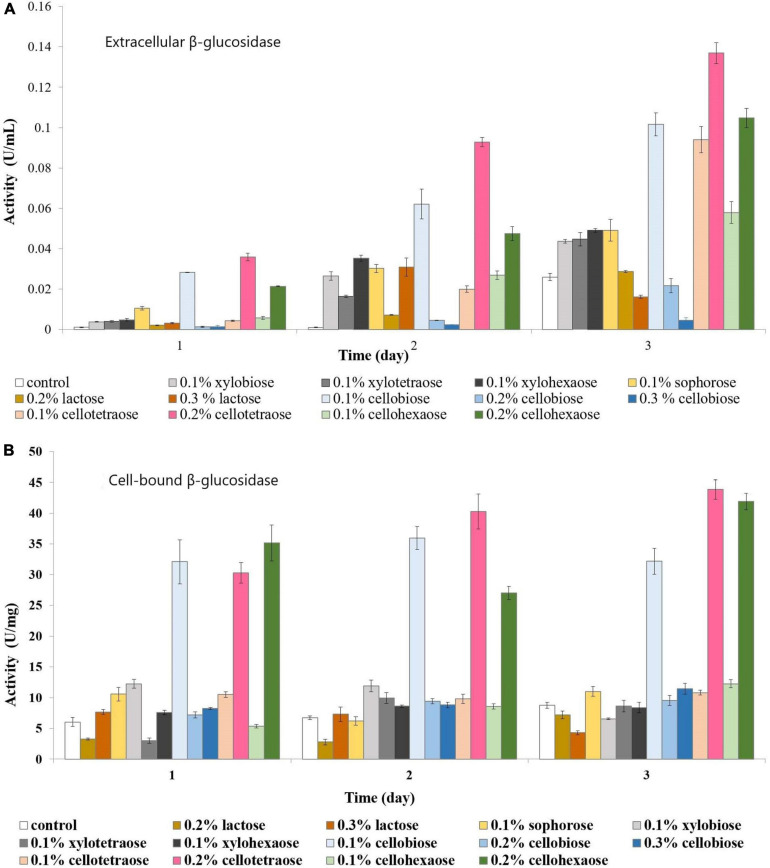
β-glucosidase activity of sucrose-grown biomass in the presence of different inducers and using pNPG as substrate. **(A)** Extracellular β-glucosidase activity; **(B)** Cell-bound β-glucosidase activity.

Both extracellular and cell-bound β-glucosidase exhibited the highest activity in the presence of 0.2% cellotetraose, followed by 0.2% cellohexaose and 0.1% cellobiose (Figures 3A,B). As with other enzymes, concentration and degree of polymerization proved to be important factors defining the efficiency of each inducer in stimulating β-glucosidase activity. Confirming previous studies on other fungi, there is elevated cell wall-bound β-glucosidase activity in this study as well (Figure 3B; Umile and Kubicek, 1986; Tiwari et al., 2013).

### Uptake Experiment Using Sucrose as Carbon Source

**FIGURE 4:**
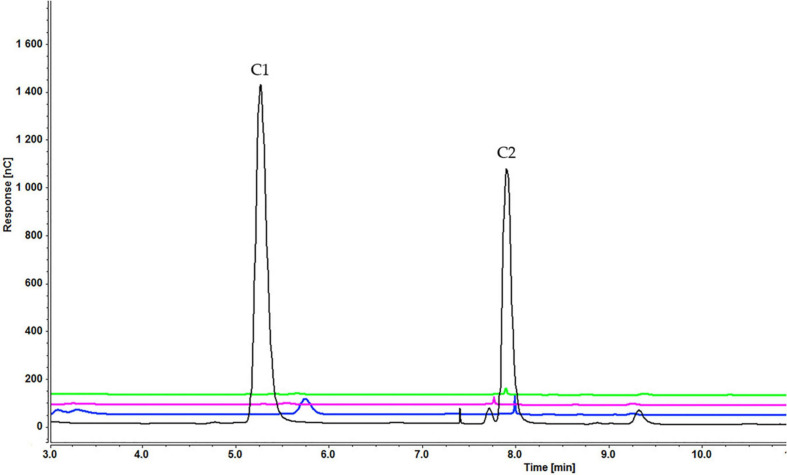
Cellooligosaccharides concentration in the medium after 1 h (black), 3 h (blue), 5 h (pink), and 7 h (green) of induction with 0.3% cellobiose (C1 = glucose, C2 = cellobiose). Figures for 0.1% (Supplementary Figure S1A) and 0.2% cellobiose (Supplementary Figure S1B) is presented in Supplementary Material.

**FIGURE 5:**
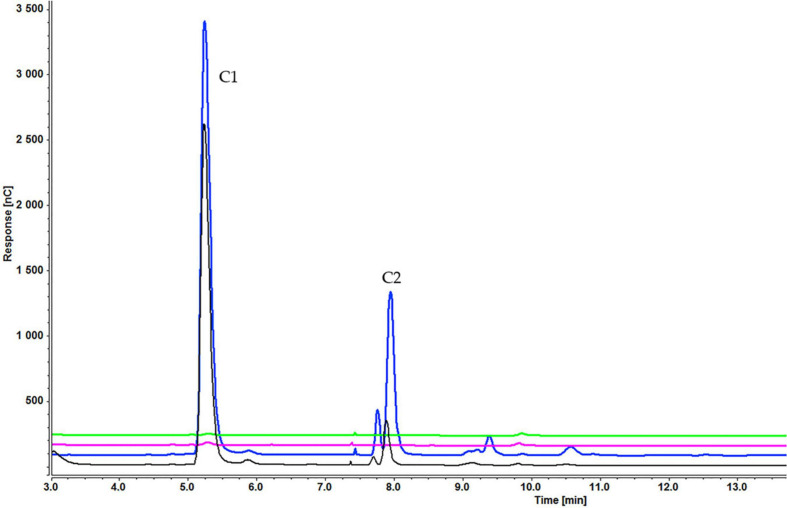
Cellooligosaccharides concentration in the medium after 1 h (black), 3 h (blue), 5 h (pink), and 7 h (green) of induction with 0.2% cellotetraose (C1 = glucose, C2 = cellobiose). The figure of 0.1% cellotetraose (Supplementary Figure S2) is presented in Supplementary Material.

**FIGURE 6:**
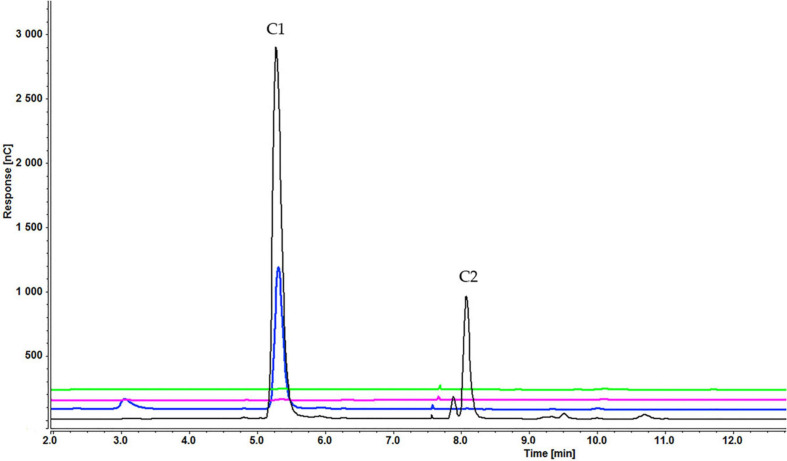
Cellooligosaccharides concentration in the medium after 1 h (black), 3 h (blue), 5 h (pink), and 7 h (green) of induction with 0.2% cellohexaose (C1 = glucose, C2 = cellobiose). The figure of 0.1% cellohexaose (Supplementary Figure S3) is presented in Supplementary Material.

**FIGURE 7:**
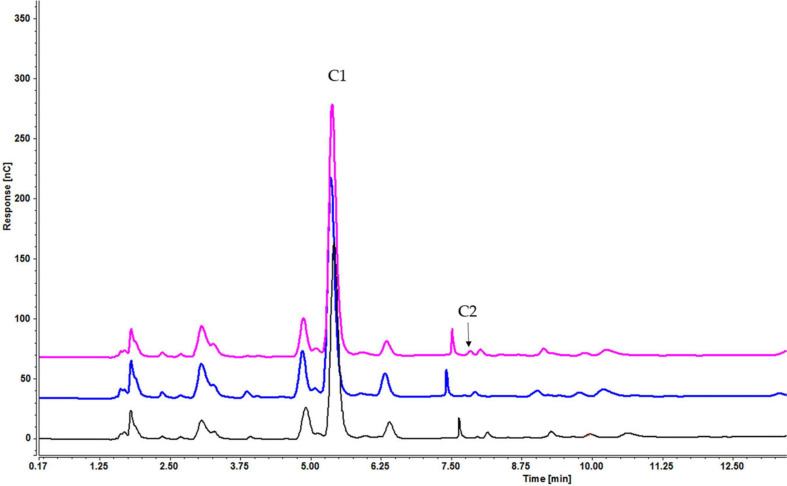
Intracellular concentration of cellooligosaccharides after 1 h of induction by cellobiose (pink), cellotetraose (black), and cellohexaose (blue) (C1 = glucose, C2 = cellobiose).

Analysis of culture medium provides information about the degradation of all cellooligosaccharides starting with the first hour of induction. Figure 4 shows that the concentration of cellobiose gradually decreased as it was degraded to glucose (Figure 4 and Supplementary Figure S1). Figures 5, 6 and Supplementary Figures S2, S3) indicate that both cellotetraose and cellohexaose were immediately degraded to glucose and cellobiose. As the latter disappeared after 3 h when cellohexaose was added, it appears that cellohexaose was degraded or was uptaken faster than cellotetraose. After 1 h of induction, glucose was detected in all intracellular samples, whereas the cellobiose peak was minimal (Figure 7). There is no sign of cellotetraose and cellohexaose inside the cell that shows either they are first degraded to smaller units and imported to the cell, or are imported and degraded quickly to cellobiose and glucose. Christakopoulos et al., showed that the initial velocity of cello-oligosaccharides degraded by *F. oxysporum* endoglucanases increased with degree of polymerization (Christakopoulos et al., 1995b,c). Supplementary Figure S4 represents peaks of pure inducers.

### Enzyme Induction Using Glycerol as a Carbon Source and Cellooligosaccharides as Inducers

**FIGURE 8:**
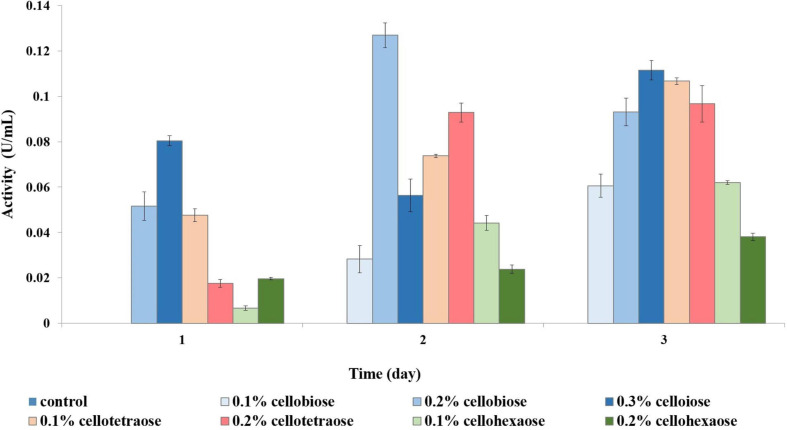
Endoglucanase activity of glycerol-grown biomass in the presence of different inducers and using CMC as substrate.

To investigate the effect of a non-carbohydrate carbon source on enzyme activity, we assessed enzymatic activity in glycerol-grown *F. oxysporum*. Growth analysis revealed that sucrose and glycerol led to a comparable amount of biomass. Unlike with sucrose-grown cells, short-chain cellooligosaccharides were better cellulase inducers when cells were grown on glycerol. For example, endoglucanase activity was the highest when cellobiose was used as inducer, followed by cellotetraose and then cellohexaose (Figure 8). Again, cellobiose concentration determined the efficiency of induction, with 0.2% cellobiose leading to the highest activity on day 2 and 0.3% cellobiose on day 3.

**FIGURE 9:**
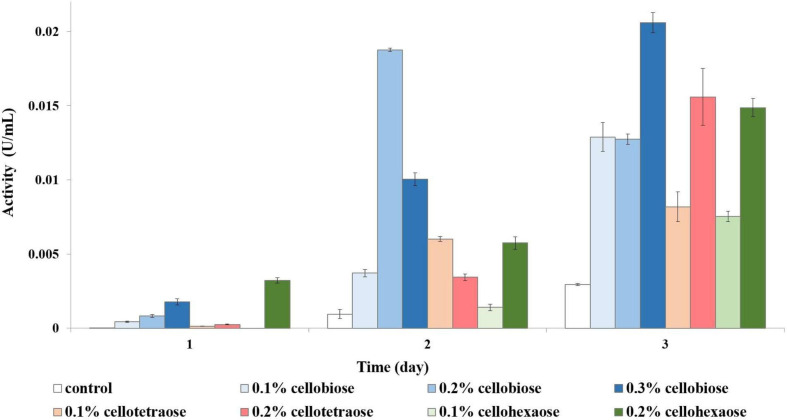
Exoglucanase activity of glycerol-grown biomass in the presence of different inducers and using pNPC as substrate.

There appeared to be a direct inverse relationship between the degree of polymerization of an inducer and its potential for exoglucanase induction. Inducers with a lower degree of polymerization were more effective, with cellobiose resulting in the highest activity, followed by cellotetraose and cellohexaose. Similarly, a higher concentration of inducer stimulated more exoglucanase activity. For example, in the case of cellobiose, 0.1% was insufficient to elicit a response, and depending on the sampling time, either 0.2 or 0.3% produced the best results (Figure 9).

**FIGURE 10:**
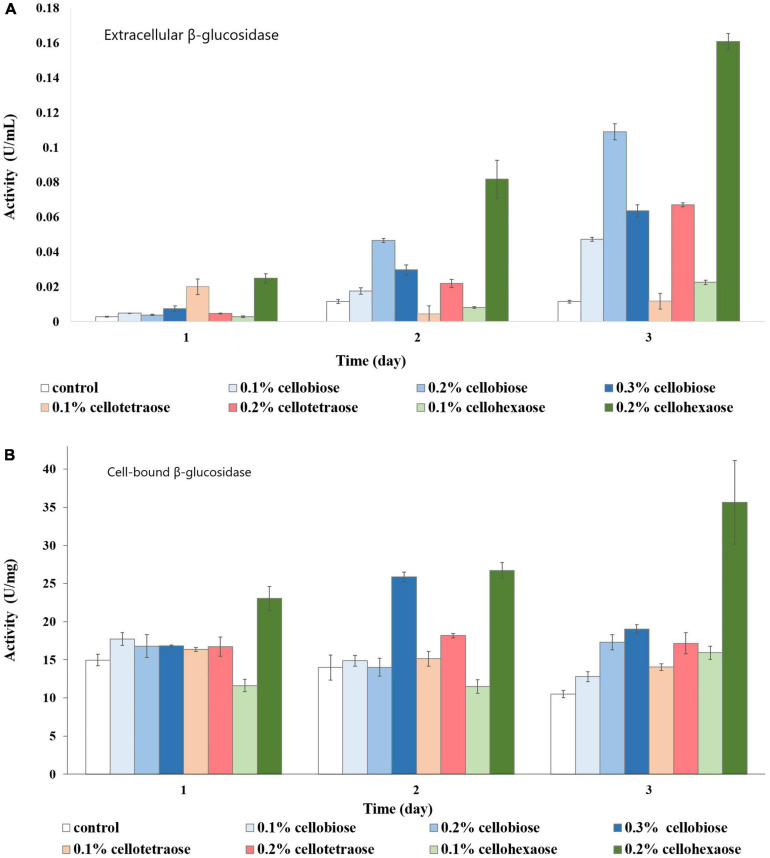
β-glucosidase activity of glycerol-grown biomass in the presence of different inducers and using pNPG as substrate. **(A)** Extracellular β-glucosidase activity; **(B)** Cell-bound β-glucosidase activity.

In the case of cell-bound β-glucosidase, most inducers except 0.2% cellohexaose and 0.3% cellobiose did not contribute to a considerable increase in activity compared to the control (Figure 10A). Even though cell-bound β-glucosidase exhibited elevated activity in all samples, including the control, it was nevertheless lower than in sucrose-grown samples, where it was clearly influenced by the added effect of invertase.

As with the cell-bound form, cellohexaose, and cellobiose proved to be better inducers of extracellular β-glucosidase activity. Here, induction efficiency depended on the concentration of the final product acting as inducer, which in turn was related to its degree of polymerization and initial concentration. Again, the best result was obtained with 0.2% cellohexaose, while the effect of 0.1% cellohexaose was negligible (Figure 10B). A concentration of 0.2% was optimal also for cellobiose. In line with previous studies (Christakopoulos et al., 1994; Olajuyigbe et al., 2016) our result confirms the elevated β-glucosidase activity of F. oxysporum, which prevents end-product inhibition. In addition, activity was slightly higher in glycerol-grown cells compared to sucrose-grown cultures.

### Uptake Experiment Using Glycerol as Carbon Source

**FIGURE 11:**
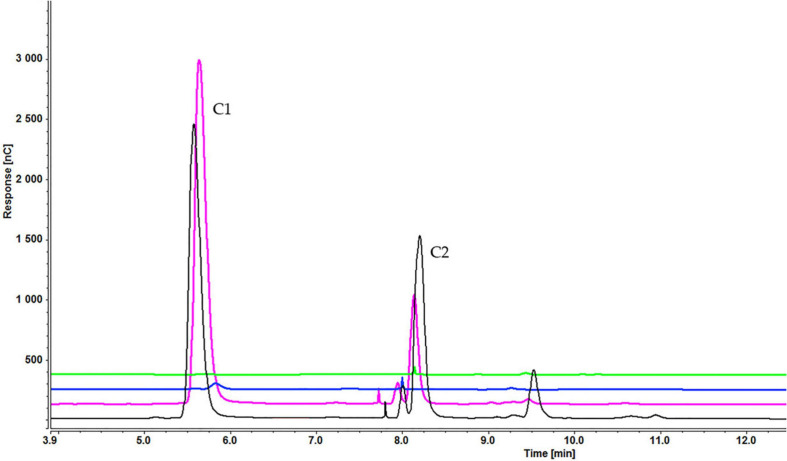
Cellooligosaccharides concentration in the medium after 1 h (black), 3 h (pink), 5 h (blue), and 7 h (green) of induction with 0.3% cellobiose (C1 = glucose, C2 = cellobiose). Figures for 0.1% (Supplementary Figure S5A) and 0.2% cellobiose (Supplementary Figure S5B) is presented in Supplementary Material.

**FIGURE 12:**
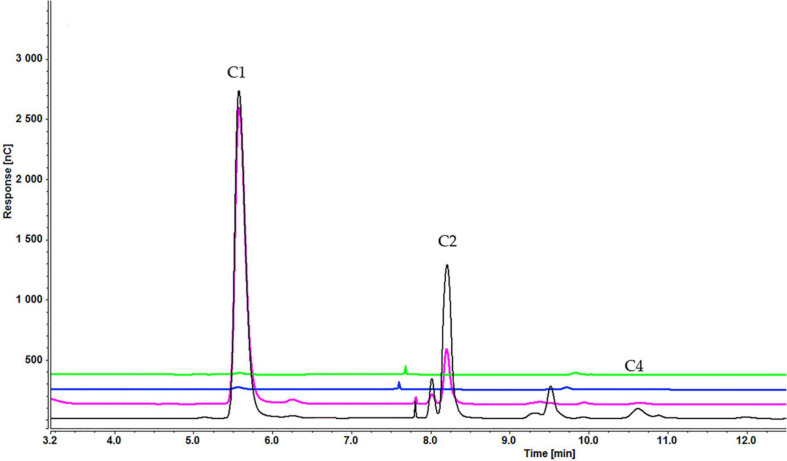
Cellooligosaccharides concentration in the medium after 1 h (black), 3 h (pink), 5 h (blue), and 7 h (green) of induction with) 0.2% cellotetraose (C1 = glucose, C2 = cellobiose, C4 = cellotetraose). The figure of 0.1% cellotetraose (Supplementary Figure S6) is presented in Supplementary Material.

**FIGURE 13:**
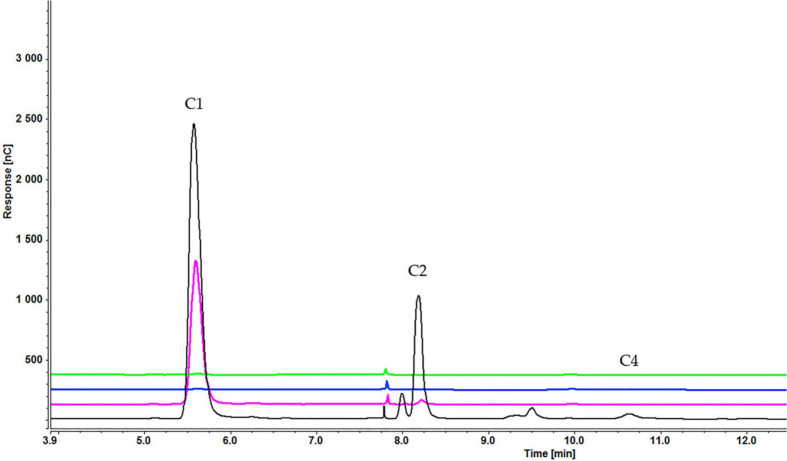
Cellooligosaccharides concentration in the medium after 1 h (black), 3 h (pink), 5 h (blue), and 7 h (green) of induction with 0.2% cellohexaose cellotetraose (C1 = glucose, C2 = cellobiose, C4 = cellotetraose). The figure of 0.1% cellohexaose (Supplementary Figure S7) is presented in Supplementary Material.

**FIGURE 14:**
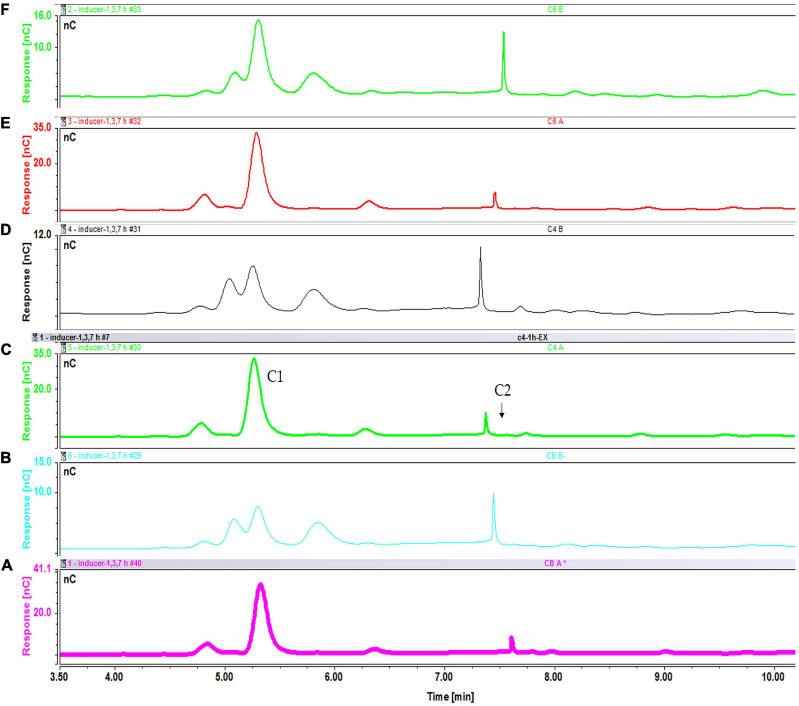
Intracellular concentration of cellooligosaccharides after 1 h of induction with cellobiose fractions A **(A)** and B **(B)**; cellotetraose fractions A **(C)** and B **(D)**; and cellohexaose fractions A **(E)** and B **(F)**.

As with sucrose-grown cells, the cellooligosaccharides uptake result for cells grown on glycerol showed the gradual degradation and consumption of inducers. Particularly, cell-bound β-glucosidases induced by glycerol can be involved in disappearance of lower concentrations of inducers. In a sample containing cellobiose, both glucose and cellobiose disappeared faster (within 1 h) when 0.1% (Supplementary Figure S5A) rather than 0.2% cellobiose (Supplementary Figure S5B) (within 3 h) and 0.3% cellobiose (5 h) was added (Figure 11). Cellotetraose was rapidly degraded to cellobiose, which was then completely consumed after 1 (0.1% cellotetraose) (Supplementary Figure S6) and 3 h (0.2% cellotetraose) of induction (Figure 12). In the case of 0.2% cellotetraose, cellotetraose was detected until the first hour of induction. Finally, when cellohexaose was used as inducer, it was degraded to cellotetraose and cellobiose within the first hour of induction, as indicated by the minuscule amount of cellotetraose detected in the 0.2% cellohexaose sample (Figure 13 and Supplementary Figure S7). Cellotetraose itself was further degraded to cellobiose, which acted as cellulase inducer. Finally, as with the sucrose experiment, intracellular cellooligosaccharides analysis revealed the presence of glucose and probably a small amount of cellobiose (Figure 14).

## Discussion

The present study indicates that the effect of different cellooligosaccharides on induction of cellulases depends on the type of carbon source utilized by the fungal culture. Accordingly, cellotetraose is more effective when *F. oxysporum* is grown on sucrose; whereas a shorter oligosaccharide such as cellobiose works better in glycerol-grown cells. Moreover, the concentration of the inducer is as important as its chain length. The carbon source used to grow the fungus may change its complement of basal enzymes irrespective of whether cellulosic material is present or not. Sucrose is the main sugar reserve in plants (Hughes and Mitchell, 1995), as well as the main substrate providing UDP-glucose for cellulose biosynthesis (Rende et al., 2017). It is also the preferred sugar consumed by plant pathogenic fungi (Hughes and Mitchell, 1995). Therefore, pathogens have developed mechanisms to optimize sucrose consumption by producing O-glycosyl-degrading enzymes such as invertases. The endo- and exoglucanase activity of samples grown on glycerol is higher than sucrose samples. This can be due to the repression of cellooligosaccharides degrading genes by sucrose, as the preferred carbon source. Investigations on filamentous fungi showed that the carbon catabolic repression by easily metabolized carbon sources such as glucose and sucrose, repress genes coding cellulases and hemicellulases even in presence of the lignocellulosic substrate (Znameroski et al., 2012). Also, there is a possibility that Stl1 (Sugar Transporter Like) transporter be influential in this increase. It is known that glycerol/H^+^ symporter encoded by *stl1* are responsible for active transport of glycerol (Ferreira et al., 2005). Previous studies including structural analysis of Stl1 transporter shows it has characteristics of sugar transporters (twelve transmembrane domains) and participates in sugar transportation, especially hexoses (Zhao et al., 1994). However, based on Ko et al. (1993), sugar transporters are not necessarily contribute in the transportation of the sugars to be metabolized and they may act as a sensor to modulate the activity or expression of the other transporters. So we assume that, in samples grown on glycerol, the higher number of Stl1 transporters can affect the cellooligosaccharides uptake and cellulase induction. Moreover, the applied inducer also influence the profile of membrane transporters and consequently enzyme induction. Carbon sources enter the cell mainly by MFS (major facilitator superfamily) transporters (Dos Reis et al., 2016). There are 17 families of MFS transporters which sugar transportation belong to families 1, 5, and 7 (Saier et al., 1999; Yan, 2013). Despite that not many of these transporters are discovered in fungi, their importance in cellulose and hemicellulose sensing and degradation in *T.reesei*, *N.crassa*, and *A. nidulans* are known (Zhang et al., 2013; Cai et al., 2014; Znameroski et al., 2014; Dos Reis et al., 2016). In *N.crassa*, CDT-1 and CDT-2 are known as “transceptors” because they are transporting receptors that participate in cellulose sensing, signaling, and cellodextrins transportation (Znameroski et al., 2014). In *A. nidulans*, CltA transport cellobiose and CltB is mainly responsible for substrate sensing and signaling (Dos Reis et al., 2016). In *T. reesei*, Stp1 which transports cellobiose and glucose is involved in carbon catabolite repression while Crt1 do not participate in transportation, instead it works for cellulolytic signaling (Zhang et al., 2013).

In this study, exoglucanase assay by using Avicel as enzyme substrate showed no activity therefore, pNPC was replaced for determining the exoglucanase activity. Avicel has a low degree of polymerization and low accessibility, which makes it a good substrate for exoglucanase (Zhang et al., 2006). However, in the absence of activity with Avicel, more sensitive substrates, such as pNPC or p-nitrophenyl-p-D-lactoside, are used. In the presence of these substrates, the exoglucanase breaks the agluconic bond between p-nitrophenyl and the disaccharide moiety to release units of cellobiose (Eriksson et al., 1990). Based on the result sophorose is not an effective inducer of *F. oxysporum* which is compatible with previous observations reporting that sophorose induced an incomplete set of cellulase enzymes (Bazafkan et al., 2014). Probably, sophorose is not the final inducer of cellulases and its signaling process does not reflect cellulose abundance (Shida et al., 2016). This study helps clarify the mechanism of cellulase production by identifying the biomass products used as preferential inducers by the fungus. Importantly, the study shows the potential of cellobiose as an efficient and cheap inducer compared to cellotetraose and cellohexaose. Understanding the molecular mechanism of cellulase induction in filamentous fungi could allow researchers to manipulate other (e.g., thermophilic) cellulolytic organisms to enable the production, purification, and examination of new lignocellulolytic enzymes (Znameroski et al., 2012). Moreover, this study illustrates that for each specific inducer, the appropriate carbon source should be selected to achieve the best outcome. The uptake experiment yielded a similar result for both carbon sources, indicating that any difference between inducers cannot be ascribed to different transporters and, in both cases, cellobiose is the final inducer. Therefore, the higher endoglucanase and exoglucanase activity of glycerol-grown samples and the different order of inducers is linked to the genes and pathways activated by sucrose or glycerol, which influence inducer sensing and transcription of cellulase-encoding genes.

## Data Availability Statement

The original contributions presented in the study are included in the article/Supplementary Material, further inquiries can be directed to the corresponding author/s.

## Author Contributions

UR and PC: conceptualization. NN, LM, UR, and PC: methodology. NN: investigation and writing—original draft preparation. LM, UR, and PC: writing—review and editing. All authors have read and agreed to the published version of the manuscript.

## Conflict of Interest

The authors declare that the research was conducted in the absence of any commercial or financial relationships that could be construed as a potential conflict of interest.
