# Supplementary material for: How Carbon Source and Degree of Oligosaccharide Polymerization Affect Production of Cellulase-Degrading Enzymes by Fusarium oxysporum f. sp. lycopersici
Source: Front Microbiol. 2021 Mar 26;12:652655. doi: 10.3389/fmicb.2021.652655 (PMC8032549; doi:10.3389/fmicb.2021.652655)
Supplement: Supplementary file 1 [file Data_Sheet_1.docx]

Supplementary Material

# Supplementary Figures

#
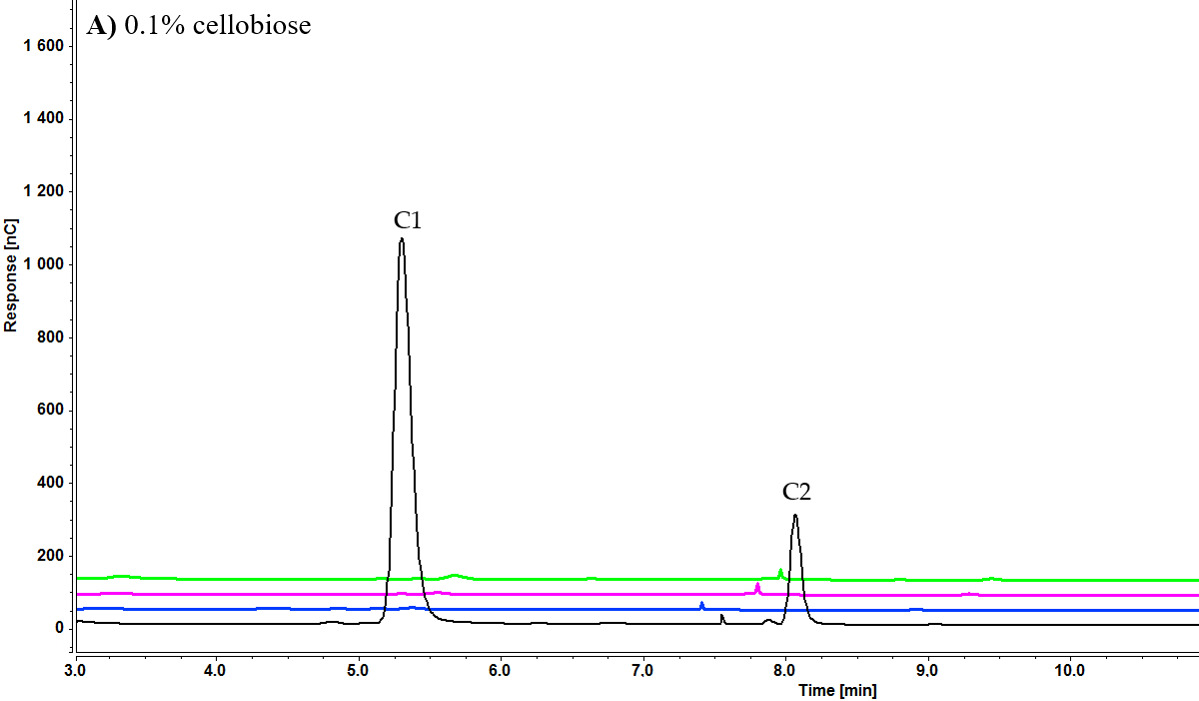


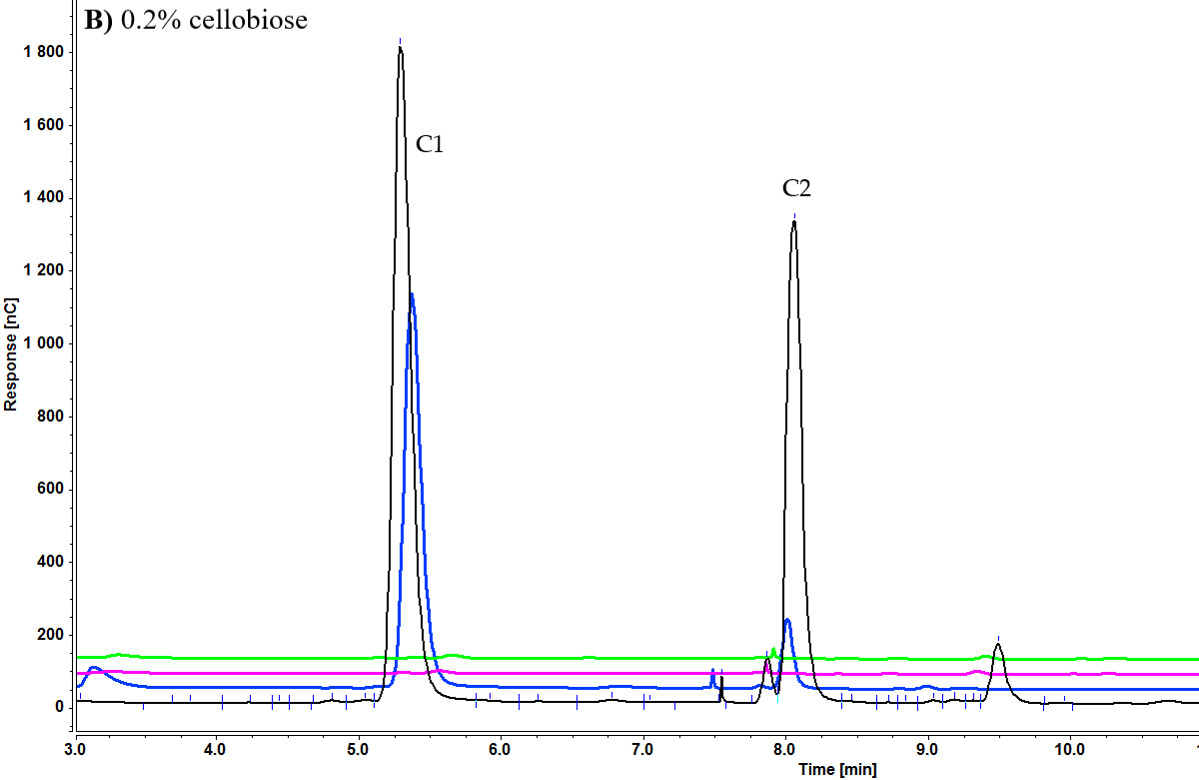


**Supplementary Figure S1.** Cellooligosaccharides concentration in the medium after 1 h (black), 3 h (blue), 5 h (pink), and 7 h (green) of induction with 0.1% cellobiose (A) and 0.2% cellobiose (B) (C1= glucose, C2= cellobiose).


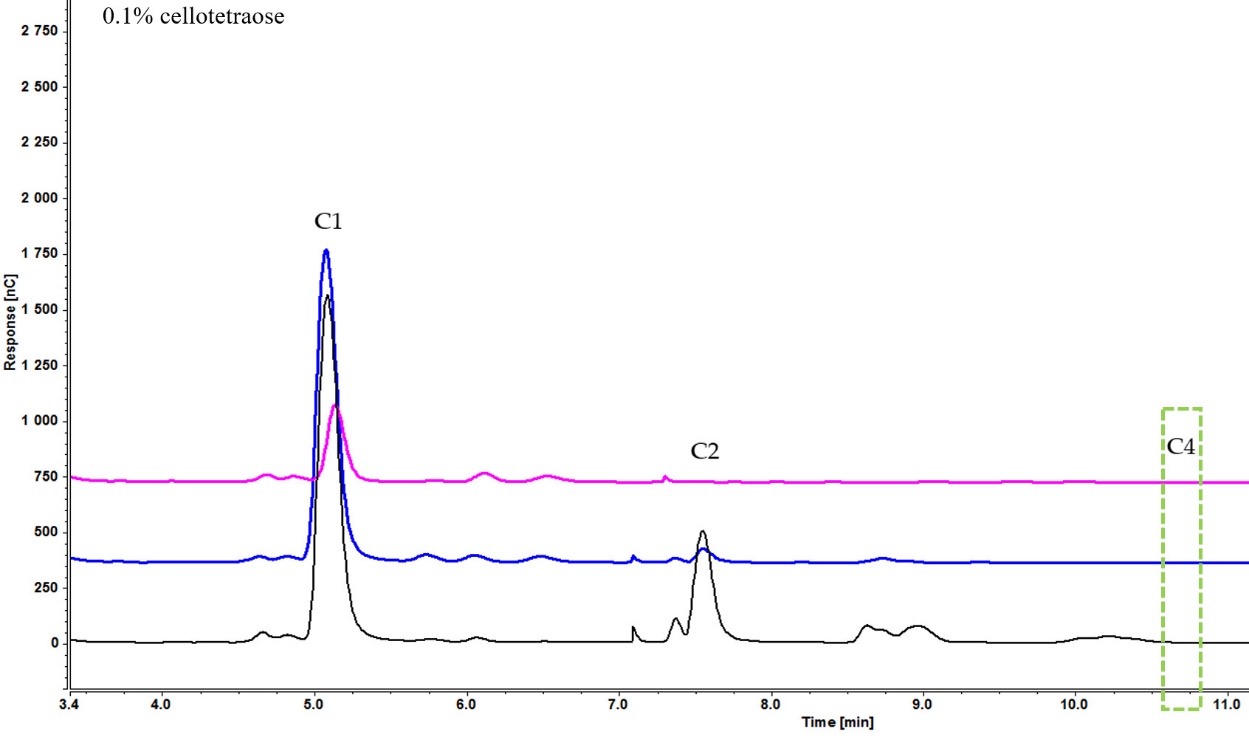


**Supplementary Figure S2.** Cellooligosaccharides concentration in the medium after 1 h (black), 3 h (blue), 5 h (pink) of induction with 0.1% cellotetraose (C1= glucose, C2= cellobiose).


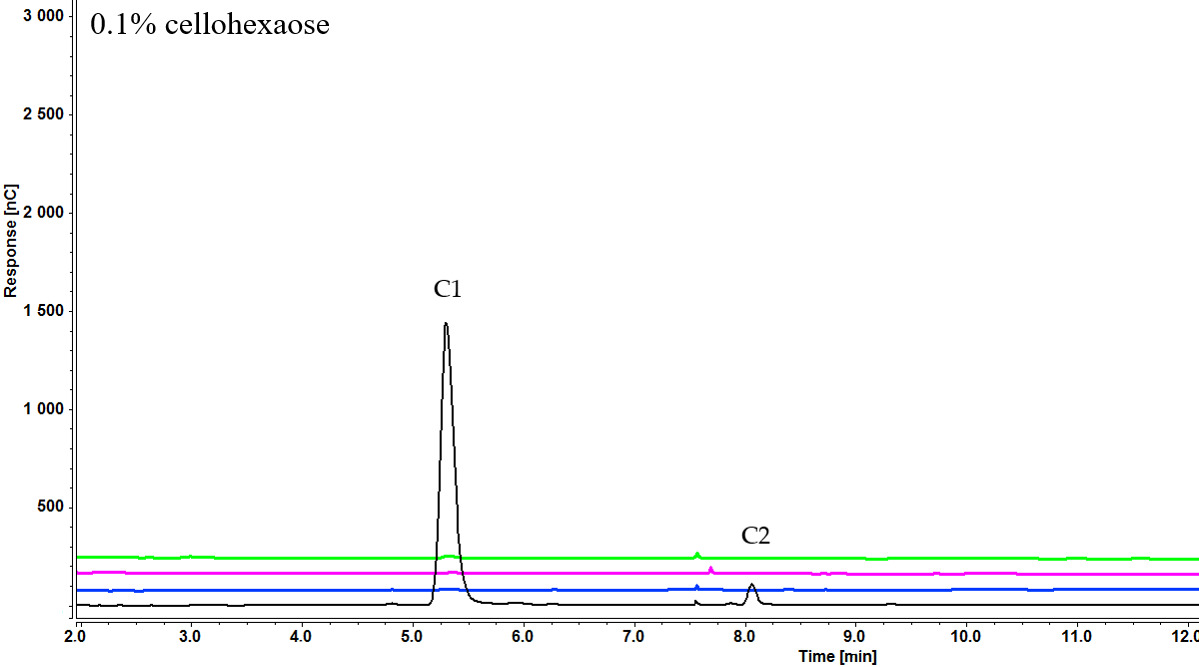


**Supplementary Figure S3.** Cellooligosaccharides concentration in the medium after 1 h (black), 3 h (blue), 5 h (pink), and 7 h (green) of induction with 0.1% cellohexaose (C1= glucose, C2= cellobiose).


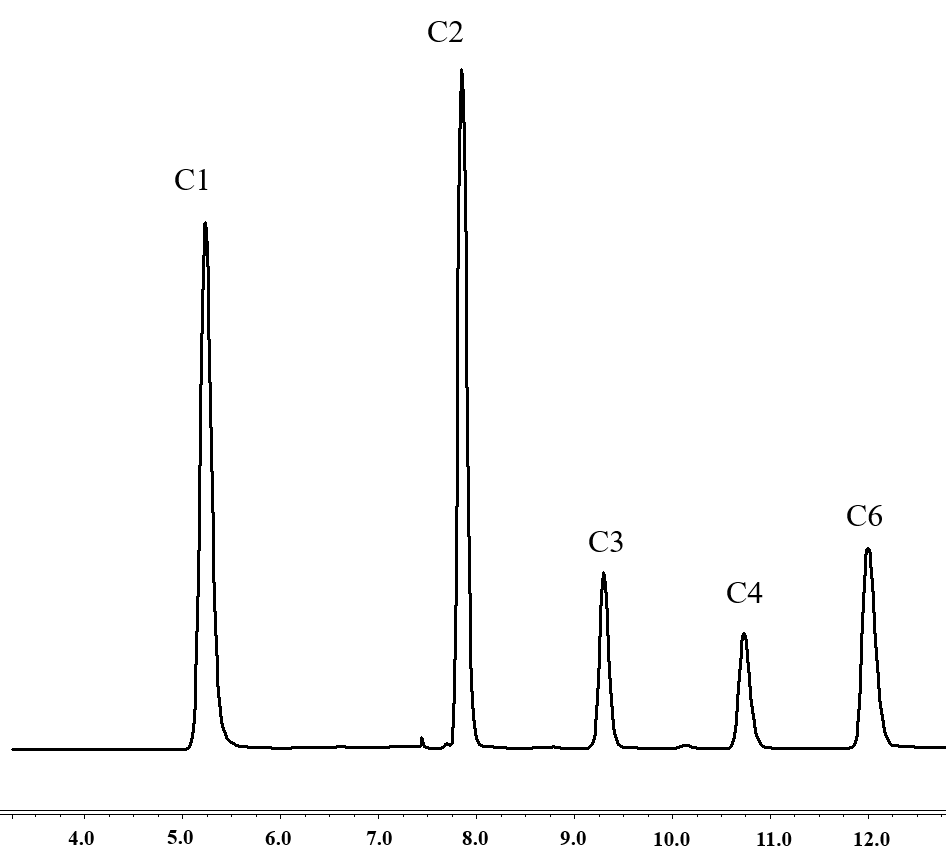


**Supplementary Figure S4.** Peaks of pure inducers (C1= glucose, C2= cellobiose, C3= cellotriose, C4= cellotetraose, C6= cellohexaose).


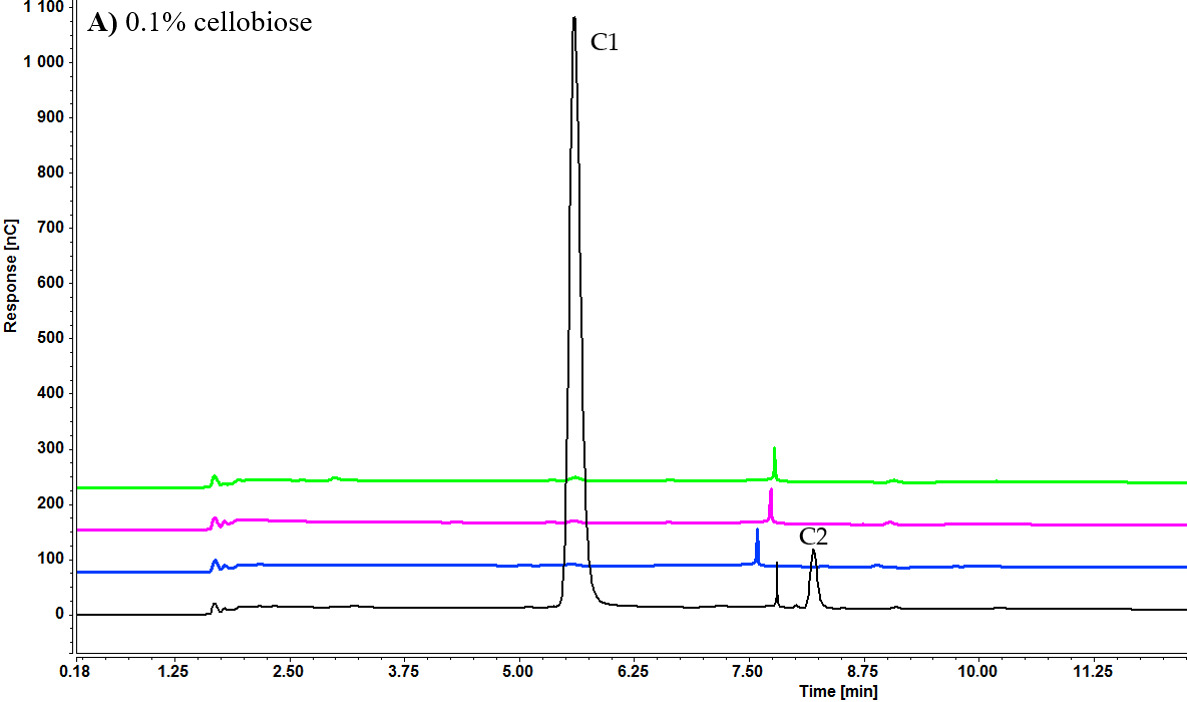


**
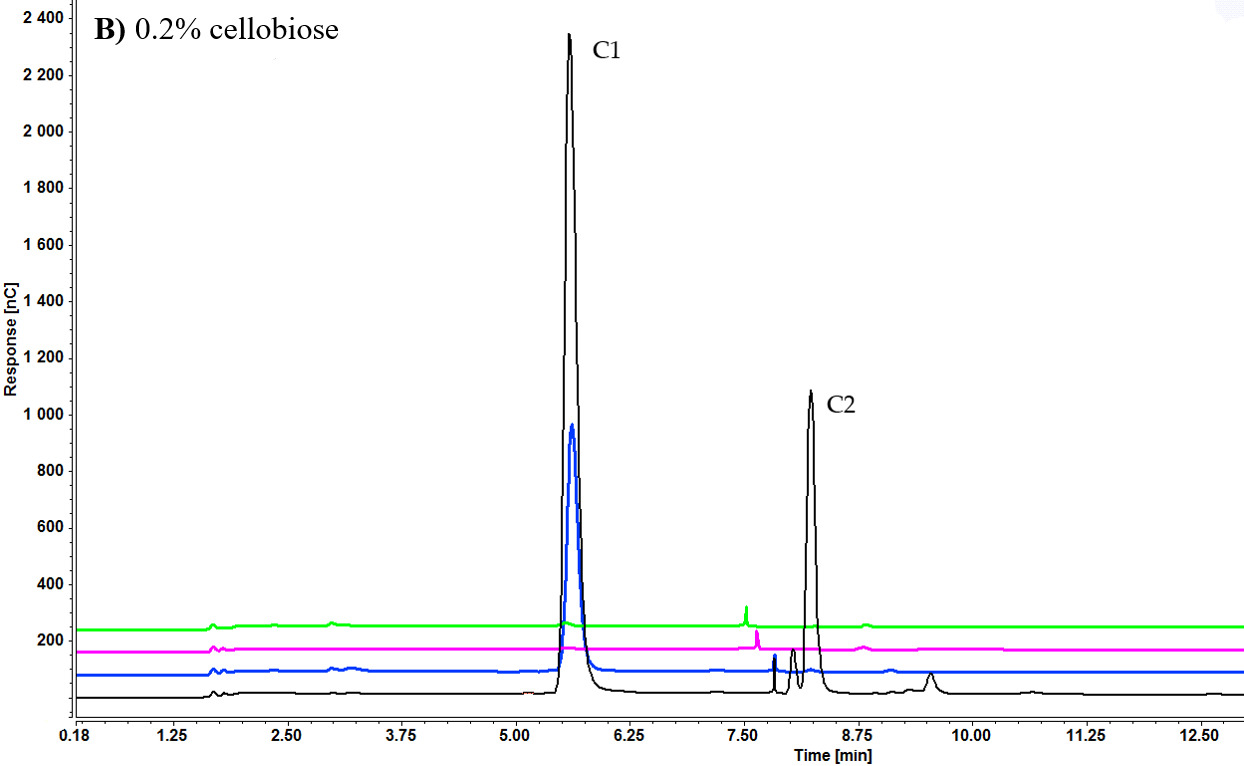
**

**Supplementary Figure S5.** Cellooligosaccharides concentration in the medium after 1 h (black), 3 h (blue), 5 h (pink), and 7 h (green) of induction with 0.1% cellobiose (A) and 0.2% cellobiose (B) (C1= glucose, C2= cellobiose).


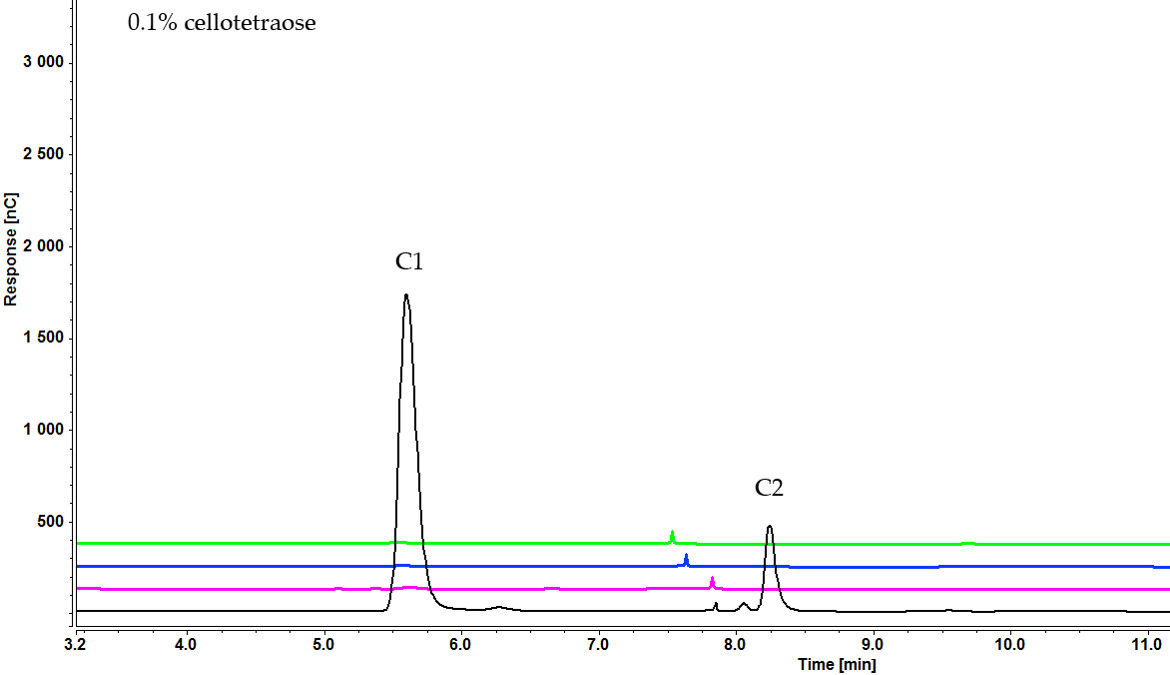


**Supplementary Figure S6.** Cellooligosaccharides concentration in the medium after 1 h (black), 3 h (pink), 5 h (blue), and 7 h (green) of induction with ) 0.1% cellotetraose (C1= glucose, C2= cellobiose, C4= cellotetraose).


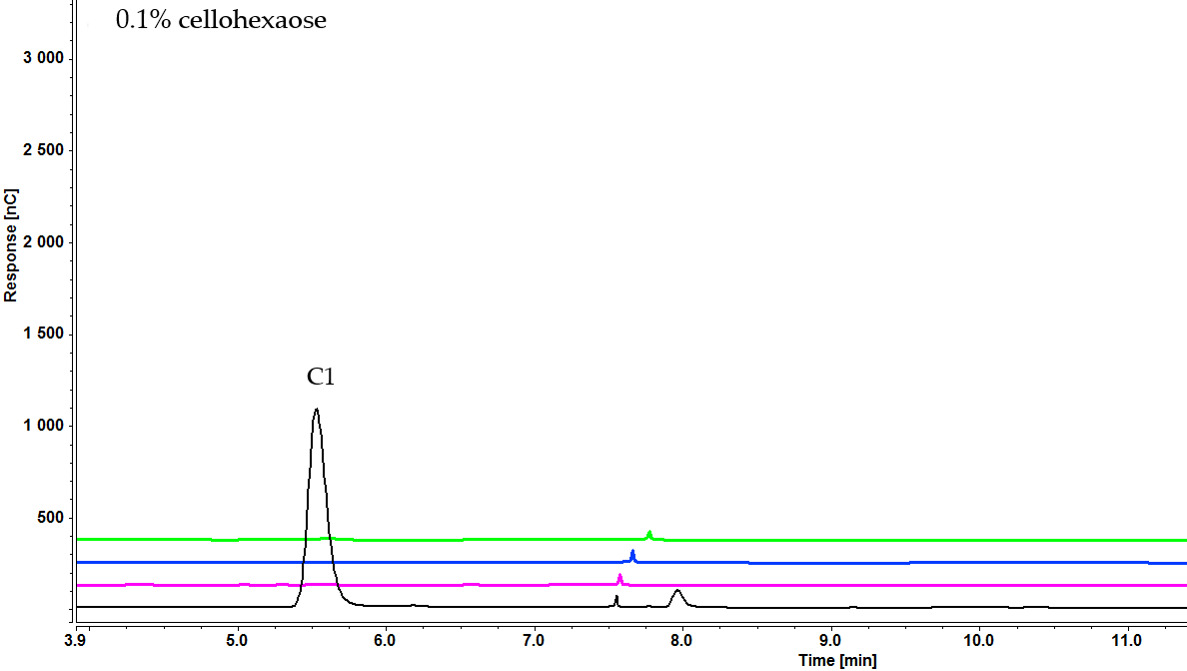


**Supplementary Figure S7.** Cellooligosaccharides concentration in the medium after 1 h (black), 3 h (pink), 5 h (blue), and 7 h (green) of induction with 0.1% cellohexaose cellotetraose (C1= glucose, C2= cellobiose, C4= cellotetraose).
